# Radiomics and deep learning for myocardial scar screening in hypertrophic cardiomyopathy

**DOI:** 10.1186/s12968-022-00869-x

**Published:** 2022-06-27

**Authors:** Ahmed S. Fahmy, Ethan J. Rowin, Arghavan Arafati, Talal Al-Otaibi, Martin S. Maron, Reza Nezafat

**Affiliations:** 1 Department of Medicine (Cardiovascular Division), Beth Israel Deaconess Medical Center and Harvard Medical School, 330 Brookline Ave, Boston, MA 02215 USA; 2 Cardiovascular Center, Tufts Medical Center, Boston, USA

## Abstract

**Background:**

Myocardial scar burden quantified using late gadolinium enhancement (LGE) cardiovascular magnetic resonance (CMR), has important prognostic value in hypertrophic cardiomyopathy (HCM). However, nearly 50% of HCM patients have no scar but undergo repeated gadolinium-based CMR over their life span. We sought to develop an artificial intelligence (AI)-based screening model using radiomics and deep learning (DL) features extracted from balanced steady state free precession (bSSFP) cine sequences to identify HCM patients without scar.

**Methods:**

We evaluated three AI-based screening models using bSSFP cine image features extracted by *radiomics*, *DL*, or combined *DL-Radiomics*. Images for 759 HCM patients (50 ± 16 years, 66% men) in a multi-center/vendor study were used to develop and test model performance. An external dataset of 100 HCM patients (53 ± 14 years, 70% men) was used to assess model generalizability. Model performance was evaluated using area-under-receiver-operating curve (AUC).

**Results:**

The DL-Radiomics model demonstrated higher AUC compared to DL and Radiomics in the internal (0.83 vs 0.77, p = 0.006 and 0.78, p = 0.05; n = 159) and external (0.74 vs 0.64, p = 0.006 and 0.71, p = 0.27; n = 100) datasets. The DL-Radiomics model correctly identified 43% and 28% of patients without scar in the internal and external datasets compared to 42% and 16% by Radiomics model and 42% and 23% by DL model, respectively.

**Conclusions:**

A DL-Radiomics AI model using bSSFP cine images outperforms DL or Radiomics models alone as a scar screening tool prior to gadolinium administration. Despite its potential, the clinical utility of the model remains limited and further investigation is needed to improve the accuracy and generalizability.

**Supplementary Information:**

The online version contains supplementary material available at 10.1186/s12968-022-00869-x.

## Background

There has been an increased use of gadolinium contrast agent (GBCA) in the magnetic resonance exams in the past two decades. It is estimated that 450 million gadolinium doses (i.e. 67 million liters) of gadolinium have been administered up to 2018 [1]. Between 1998 and 2008, GBCA use increased tenfold [2]. In addition to cost, excess use of gadolinium has caused environmental contamination. GBCA are predominantly excreted unmetabolized via the kidneys and enter aquatic ecosystems [3]. A recent study showed gadolinium contamination of tap water-based beverages sold in fast food chains in Germany [4]. There has also been a concern regarding the safety of increasing anthropogenic gadolinium found in water surrounding major cities [5]. GBCA is generally considered safe, except in patients with impaired kidney function who are at high risk of developing nephrogenic systemic sclerosis (NSF) [6, 7]. These patients are often excluded from magnetic resonance imaging exams with GBCA or require hemodialysis with close monitoring after GBCA administration. Beyond NSF, emerging evidence has shown GBCA deposition in various organs after repeat GBCA administration [8–10]. Although there is no clear evidence that GBCA retention causes any clinical symptoms, GBCA should be used judiciously, and healthcare providers should engage in shared decision-making with patients using an individualized risk–benefit assessment.

Over the past two decades, there has also been an exponential growth in use of cardiovascular magnetic resonance (CMR) imaging. GBCA is commonly used in CMR for evaluation of vascular anatomy, myocardial perfusion, and late gadolinium enhancement (LGE) for scar imaging. While there have been significant efforts in contrast-free angiography techniques, there are only limited alternatives to LGE scar imaging. Conventional non-GBCA (or native) CMR imaging sequences such as magnetization transfer [11], cine balanced steady state free precession (bSSFP) [12], and T_1_ mapping [13] have been used to image myocardial scars but are limited by low scar-to-normal myocardium contrast [11, 13, 14]. More recently, deep learning (DL) analyses have been used to synthesize LGE image contrast from non-GBCA images [15, 16]. In particular, deep convolutional neural networks (CNN) were used to automatically extract local image features from conventional cine images [15] or combined cine images and native T_1_ maps [16] to generate images mimicking LGE. Large datasets of paired native and LGE images at the same anatomical cross-sections were necessary to train these CNN models.

Despite the potential of non-GBCA CMR viability imaging, LGE remains the clinical gold-standard for assessing viability and identification of myocardial scarring. Instead of trying to replace LGE with a non-contrast scar imaging, reducing the use of GBCA can be achieved through limiting LGE scans to patients with high likelihood of having scar. This approach (which we adopt in this study) depends on identifying patients without scar through post-processing of currently available non-GBCA imaging sequences. This approach can be particularly beneficial to hypertrophic cardiomyopathy (HCM) patients, who are usually young and subject to receiving multiple GBCA doses although the prevalence of HCM scars is less than 50% [17] 18. Several studies recently showed the potential of identifying patients with scar using radiomic analysis of non-GBCA T_1_ maps [19] or cine images [20, 21]. Radiomics based analyses are based on extracting a large number of *handcrafted* mathematical quantities representing the image texture of the myocardium in the native images [22]. Radiomic features encode higher-order statistics about the spatial distribution of the image intensities and thus are much richer in information-content compared to raw pixel intensity values. Different machine learning algorithms, such as logistic regression [21], decision trees [19], and extreme gradient boost [20] classifiers, were used to learn the association between the radiomic features and presence of scar. Despite the potential of radiomics, using hundreds of highly-correlated handcrafted features may not be the optimal approach to represent myocardial scarring.

Deep learning (DL) based image analysis represents an alternative approach for reliable extraction of local and global image features. Several studies showed remarkable success in diagnosing different diseases through medical image classification based on DL features [23–25]. DL features are extracted by a large number of image filters. The parameters of these DL filters are estimated using a training image set such that the extracted features result in optimal image classification.

Although data-driven extraction of DL features thought to be more powerful than feature handcrafting in radiomics [26, 27], there is a limited data on their potential to detect myocardium scars in non-GBCA CMR sequences [28]. Also, it is not clear whether radiomics and DL features are redundant or complementary. In this study, we develop and compare three approaches for extracting bSSFP image features to identify HCM patients without scar: radiomics; DL; and combined DL-radiomics analyses. We hypothesize that radiomics and DL features are not necessarily fully correlated and that a combination of radiomics and DL features can improve the discrimination between HCM patients with vs without myocardium scars.

## Methods

### Study population

**Fig. 1:**
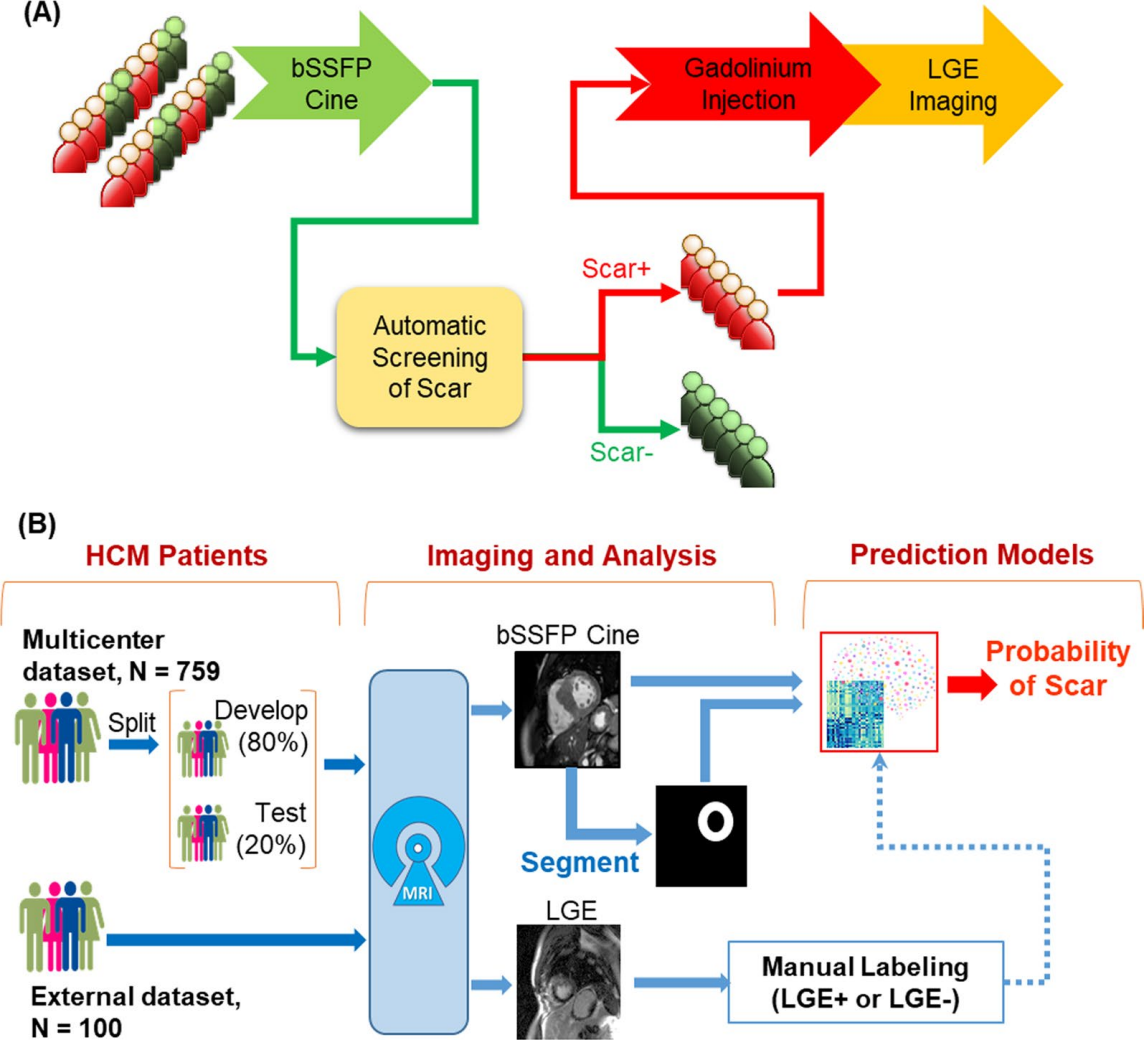
The proposed cardiovascular magnetic resonance (CMR) workflow for hypertrophic cardiomyopathy (HCM) cine datasets (**A**) and study design (**B**) for reducing unnecessary late gadolinium enhancement (LGE) scans

The workflow proposed for identifying patients without scar and triage them from LGE scans is depicted in Fig. 1a. To develop and evaluate scar prediction models, we retrospectively collected two datasets for HCM patients: internal and external (Fig. 1b). The internal dataset included 993 HCM patients from a multi-center HCM study [17] and was used for model development (i.e., training and validation) and held-out internal testing. The dataset was collected from 2003 to 2018 and included scans from 7 imaging centers: Tufts medical center (Boston, Massachusetts, USA), Minneapolis Heart Institute (Minneapolis, Minnesota, USA), Toronto General Hospital (Toronto, Canada), and four medical centers in Florence, Rome, Genova, and Bologna (Italy). Two readers reviewed the LGE images of the internal dataset, and we excluded cases where there was no agreement on presence of scar, e.g., due to low image quality or artifacts. The resulting dataset with consensus readings included 759 patients from the 7 centers (Tufts, n = 321; Minneapolis, n = 125; Toronto, n = 141; Italy, n = 172) and was split (patient-wise) into development (n = 600) and held-out testing (n = 159) cohorts. Five-fold cross-validations were employed to split the development dataset into training (80%) and validation (20%) subsets for determining the optimal model parameters. Stratified patient-wise random sampling was used to maintain same number of patients with and without scar in the testing subset. The testing subset is referred to as internal testing set because it is sampled from the same cohort used for model development. An external testing dataset for 100 HCM patients acquired at Beth Israel Deaconess Medical Center (BIDMC, Boston, Massachusetts, USA) from 2004 to 2014 was used to test the generalizability of the developed models. Both internal and external testing datasets were held-out (i.e., not seen by the models) during training or validation phases. The use of patient datasets for research purpose was approved by the Institutional Review Board (IRB) of the participating institutions.

### Image acquisition

All scans were performed using the same imaging protocol on 1.5 T CMR scanners from three major vendors: Philips Medical Systems, Best, The Netherlands (Achieva, n = 424); General Electric Medical Systems, Waukesha, Wisconsin, USA (total, n = 138; Signa Genesis, n = 11; Signa Excite, n = 127), Siemens Healthineers, Erlangen, Germany (total, n = 197; Sonata, n = 5; Avanto, n = 62; Symphony, n = 1; Verio, n = 3; NA, n = 126). The external testing dataset was acquired on 1.5 T CMR scanner (Achieva, Philips Healthcare). Each patient dataset included breath-hold electrocardiogram (ECG)-gated bSSFP cine sequences of short-axial slices acquired with the following parameters: TR = 2.5–3.6 ms, TE = 1.1–1.7 ms, flip angle = 39°–60°, 17–30 cardiac phases, pixel resolution = 0.6–1.4 mm, slice thickness = 8–10 mm, slice gap = 8–10 mm, and bandwidth = 920–1150 Hz. In addition to bSSFP cine images, each patient dataset included LGE dataset that was used only for identifying presence of myocardial scar as described below.

### Image preparation and labeling

From each patient’s dataset, we extracted non-GBCA bSSFP cine and LGE at matched anatomical slices. The left ventricle borders were automatically delineated in the short-axial cine slices using cvi42 image analysis package (version 5.10., Circle Cardiovascular Imaging Inc., Calgary, Canada). The automatically generated contours were then manually reviewed and corrected, if needed, by an experienced operator (6 years of CMR experience). For each cine slice location, only two timeframes: end-systole and end-diastole, were manually selected and used for analysis. All images were resampled to an in-plane resolution 1 × 1 mm^2^ and normalized to a fixed size (256 × 256) and fixed intensity range (from 0 to 1). For DL, the images were automatically cropped to size 128 × 128 around the image center to reduce computational and memory requirements.

Two independent readers (3 and 6 years CMR experience) evaluated the LGE scans and labeled each patient as LGE + (i.e., with scar) or LGE − (i.e., no scar). A patient was labeled LGE + if scar was identified in one or more LGE slices, otherwise the patient is labeled LGE −. For the testing datasets, a reader (E.R.) with 7 year CMR experience in HCM patients reviewed the LGE data and confirmed presence/absence of scar.

In the development image subset, we mitigated the imbalance between scar vs no-scar images by using only 6 mid-ventricular cine slices from LGE− patients and only the slices with scar from LGE + patients. In the testing dataset, we used all available slices because, in practice, patients with or without scar are not known a priori. The total number of images used for developing and testing the scar identification models was 3790 and 1063 images, respectively.

### Radiomics feature extraction

We used the Pyradiomics open-source library (version 3.0.1) [29] to extract the cine image radiomics features of the myocardium. All types of 2D radiomics features available in the Pyradiomics library were computed for each cine image as well as 9 derived versions (i.e., total 10 images). The latter were obtained by passing the cine image through 9 different filters: gradient, logarithmic, squaring, square-root, exponential, and 4 wavelet-transform filters. The extracted features included: (1) 14 shape parameters such as area and maximum diameter of the myocardium; and (2) 93 texture features including statistical parameters computed for the histogram, gray-level run-length matrix (GLRLM), gray-level co-occurrence matrix (GLCM), and local binary patterns (LBP). In total, the number of extracted features per cine image was 944 features (10 filtered images × 93 texture features/image + 14 shape features extracted from the myocardium contours). All features were normalized to a unity range from zero to one.

### Radiomics analysis

To reduce the redundancy among the extracted radiomics features, we used least absolute shrinkage and selection operator (LASSO) regression algorithm to select the features that are most important for identifying scar. First, we randomly selected a validation subset (20%) from the development dataset to evaluate the power of identifying myocardium scars using 5, 10, and 20 features. A set of ten features yielded the best performing model, where performance was measured by the area under the curve, AUC, of the receiver operating characteristics, ROC, averaged over a fivefold cross-validations. We used logistic regression (LR) classifier with L1-norm penalty and balanced class-weight to classify each image (represented by the selected 10 radiomics features) into LGE + and LGE −. The LR classifier was trained and tested using the development and testing subsets, respectively. The output of the classifier was the probability of scar in the input image, P_Radiomics_(Scar | *x*).

### Deep learning analysis

We developed a DL model to estimate the probability of scar in non-GBCA cine images. The model input was a 3D image (128 × 128 × 3), with the third dimension contained the cine image, myocardium mask, and their multiplication. The model output was the probability of scar in the input image, P_DL_(Scar | *x*). The developed model was comprised of two sub-networks: CNN and fully-connected network (FCN) trained end-to-end. The hyperparameters of the model; namely, number of channels per layer, learning rate, convolutional kernel size, and dropout rate, was selected using hyperband optimization algorithm [30]. We arbitrary set the other parameters such as number of convolutional blocks, order of functional layers, loss function, optimization algorithm, etc. The final parameters were as follows: The CNN module included seven functional blocks composed of one convolutional layer (kernel size = 5 × 5) followed by a ReLU activation function and a maximum-pool layer (size = 2 × 2). The FCN subnetwork was composed of three consequent dense layers with number of nodes = 1024, 256, and 1, respectively. A dropout layer (with probability = 50%) was added after each of the first two FCN dense layers. Both the CNN and FCN subnetworks were connected back-to-back (coupling was achieved through a tensor flattening layer) to form a single DL model. Training of the model was performed using the following parameters: minibatch size = 8, learning rate = 0.0001, binary cross-entropy loss function, Adam optimizer. To further reduce chances of overfitting, we used image augmentation inline with CNN model training through random image flipping, shearing (from 0 to 20%), zooming (from 0 to 20%), and rotation (from − 30° to 30°). The model with the best performance, measured by the average accuracy of scar identification over the validation subset, was stored and used to evaluate the performance on the testing datasets. We used gradient-weighted class activation map (Grad-CAM) algorithm to display a heatmap representing the image regions contributing most to the model prediction. We passed the third convolutional layer in our model to Grad-CAM to allow generating heatmaps with an adequate spatial resolution [31].

### Combined radiomics and deep learning based cine analysis

**Fig. 2:**
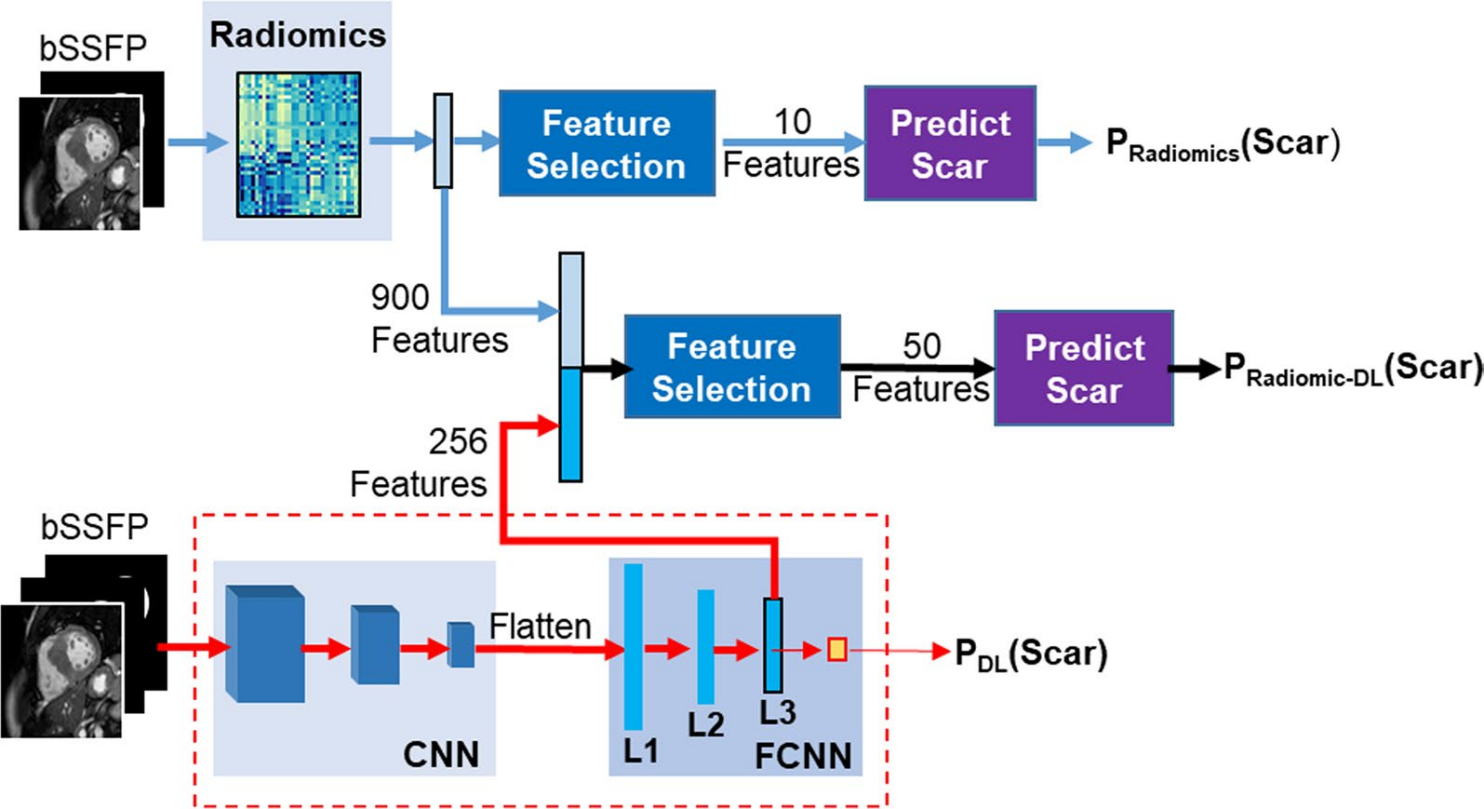
Development of three scar prediction models: radiomics, deep learning (DL) and combined DL-Radiomics. Activations from layer L3 of the fully connected neural network (FCNN) are extracted and used to build the DL-Radiomics model. *CNN*  convolutional neural network

To build the combined DL-Radiomics model, we extracted a set of DL features from the pre-trained DL model described above. First, each image in the dataset was passed through the model and the activation of the second-to-last FCNN layer (L3 in Fig. 2; n = 256 nodes) was stored and used as the image DL features. We note that training and testing datasets were kept separate to prevent leakage of information from the testing dataset into the DL-Radiomics model. The DL features (n = 256) were then concatenated with the radiomics features (n = 944) to create a set of combined DL-radiomics features (n = 1200) for each image. To determine the optimal number of features, we randomly split the development dataset into training (80%) and validation (20%). We trained several models with different number of parameters (from 5 to 50) and used the validation subset to evaluate the performance (measured by average AUC over fivefold cross-validations). The optimal number of features was 20, which was then used to develop the final DL-Radiomics model. The selected sets of the DL and radiomic features were input to a classifier trained to merge the DL and radiomic features and output the probability of scar, P_DL-Radiomics_(Scar | *x*). To determine the best classifier, we used the development dataset (split into 80% training and 20% validation) to develop and evaluate 5 different classifiers; namely, LR, multi-layer NN, random-forests, gradient boosted decision trees, and support vector machines (SVM). An LR classifier (with L1-norm penalty and balanced class-weight) resulted in the best AUC (i.e., highest average and lowest standard deviation over fivefold cross-validations), which was then used to develop the final model. Details of the performance of the different classifiers are available in Additional file 2: Table S1. Model implementation is publicly available (https://github.com/HMS-CardiacMR/DL-Rad-ScarPrediction).

### Reproducibility analysis

We tested the reproducibility of the developed models by repeating the process of building and testing the scar prediction models using fivefold cross-validations. In each repetition, the development dataset was split into training and validation subsets and the process of feature extraction and selection was repeated for all three pipelines. This yielded 5 trained versions for each of the Radiomics, DL, and combined DL-Radiomics models. The cross-validations were performed only on the development dataset; that is the same testing cohort of patients was used to evaluate the different versions of the developed models.

### Statistics and data analysis

Patients’ characteristics were summarized as number (percentages) or mean (standard deviation) for categorical and continuous variables, respectively. Two-sided t-test or Mann–Whitney U-test was used for comparing continuous variables as appropriate. For comparison of categorical data, the Pearson Chi-squared test was used. DeLong’s test was used to compare AUC of the different models. Statistical analyses were performed using a python script (scipy-stat statistical module, version 1.6.2) or Matlab (Mathworks Inc., Natick, Massachusetts, USA). All tests were two-sided with significance level = 0.05. A patient was labeled LGE + if at least one cine image had *P*_*f*_ (Scar | *x*) > T_cutoff_, where T_cutoff_ is an arbitrary threshold used to control the sensitivity of the model to presence of scar. Because the developed models are used to screen patients for scar, they need to operate at high sensitivity level to avoid misclassification of patients with scar. Therefore, we set T_cutoff_ to a value corresponding to a sensitivity of at least 90%. Additionally, having all models operating at the same sensitivity level allowed fair comparison of the performance of the different models. We note that model specificity represents the subset of patients without scar who are correctly identified by the model. The model with best AUC computed on the internal testing dataset was used as the final model applied to the external testing dataset.

## Results

Clinical and demographic data for the internal and external cohorts are summarized in Tables 1 and 2, respectively. In general, patient characteristics spanned a wide range of values indicating a heterogeneous cohort of HCM patients. In the internal cohort, the mean left ventricular (LV) mass (LVM), LV end-diastolic volume (LVEDV), and LV ejection fraction (LVEF) were 158 ± 58 g, 147 ± 40 ml, and 68 ± 10%, respectively. In the external cohort, LVM, LVEDV, and LVEF were 161 ± 56 g, 152 ± 37 ml, and 65 ± 8%, respectively. In the internal cohort, patients with and without scar showed significantly different measurements for LVEDV and LV end-systolic volume (LVESV), LVEF, LV stroke volume, LVM, and LV max wall thickness (Table 1). In the external dataset, LVM was the only significantly different parameter between patients with and without scar (Table 2). Furthermore, manual reading of the LGE images identified myocardium scar in 276 patients (36% of 759 patients) in the internal cohort versus 57 patients (57% of 100 patients) in the external testing dataset.

**Table 1:** Patient characteristics in the internal dataset grouped by presence of late gadolinium enhancement (LGE) myocardium scar

	All patients (n = 759)			Development cohort (n = 600)			Internal testing cohort (n = 159)		
	All	LGE + (n = 276)	LGE− (n = 483)	All	LGE + (n = 201)	LGE− (n = 399)	All	LGE + (n = 75)	LGE− (n = 84)
Male	504 (66%)	210 (76%)	294 (57%)	387 (63%)	152 (76%)	235 (59%)	117 (74%)	58 (77%)	59 (70%)
Age (years)	50 ± 16 (52)	49 ± 16 (52)	51 ± 16 (53)	51 ± 16 (52)	50 ± 16 (51)	51 ± 17 (53)	50 ± 15 (52)	48 ± 16 (52)	51 ± 15 (52)
Weight (kg)	84 ± 18 (82)	85 ± 18 (82)	84 ± 17 (82)	84 ± 18 (82)	85 ± 19 (82)	83 ± 17 (81)	84 ± 15 (38)	83 ± 15 (80)	86 ± 16 (82)
BMI (kg/m^2^)	43 ± 14 (39)	44 ± 19 (39)	43 ± 18 (39)	43 ± 19 (39)	45 ± 21 (40)	42 ± 18 (39)	43 ± 16 (38)	41 ± 15 (37)	44 ± 16 (39)
LVEDV (ml)	147 ± 40 (143)	155 ± 38 (151)	142 ± 40 (140)	146 ± 41 (142)	156 ± 40 (151)	141 ± 40 (139)	149 ± 35 (149)	152 ± 32 (151)	147 ± 37 (146)
LVESV (ml)	48 ± 22 (43)	54 ± 26 (48)	44 ± 18 (41)	47 ± 22 (42)	53 ± 27 (47)	44 ± 19 (41)	50 ± 21 (46)	54 ± 24 (49)	46 ± 17 (42)
SV (ml)	99 ± 29 (95)	102 ± 27 (96)	97 ± 29 (94)	99 ± 29 (94)	103 ± 28 (97)	97 ± 29 (93)	99 ± 26 (96)	98 ± 25 (94)	100 ± 28 (99)
LVEF (%)	68 ± 10 (69)	66 ± 11 (68)	69 ± 9 (70)	68 ± 10 (11)	66 ± 10 (68)	69 ± 10 (70)	67 ± 10 (69)	65 ± 11 (66)	69 ± 8 (71)
CO (l/min)	7 ± 4 (6)	7 ± 4 (6)	7 ± 4 (6)	7 ± 3 (6)	7 ± 2 (6)	7 ± 4 (6)	7 ± 5 (6)	7 ± 6 (6)	7 ± 2 (7)
LVM (g)	158 ± 58 (150)	185 ± 61 (172)	142 ± 49 (136)	156 ± 58 (149)	188 ± 63 (176)	141 ± 48 (135)	162 ± 58 (152)	177 ± 58 (160)	149 ± 54 (139)
LV-MWT (mm)	18 ± 5 (18)	21 ± 5 (21)	16 ± 4 (16)	18 ± 5 (18)	21 ± 5 (21)	16 ± 4 (16)	18 ± 5 (18)	21 ± 5 (19)	16 ± 4 (16)
LGE presence	276 (36%)	276 (100%)	0	201 (33%)	201 (100%)	0	75 (47%)	75 (100%)	0

Data is represented as n (%) or mean ± SD (median)

*BMI* body mass index, *CO* cardiac output, *LV* left ventricle, *LVEF*  LV ejection fraction, *LVEDV* left ventricular end-diastolic volume, *LVESV* left ventricular end-systolic volume, *LVM*  LV mass, *LGE*  late gadolinium enhancement, *MWT*  maximum wall thickness, *SV*  stroke volume

**Table 2:** Patient characteristics in the external dataset grouped by presence of late gadolinium enhancement (LGE) myocardium scar

	All (n = 100)	LGE + (n = 57)	LGE− (n = 43)
Male	70 (70%)	42 (74%)	28 (65%)
Age (years)	53 ± 14 (55)	51 ± 14 (52)	56 ± 14 (56)
Weight (kg)	85 ± 20 (84)	86 ± 20 (83)	85 ± 21 (86)
BMI (kg/m^2^)	43 ± 12 (42)	43 ± 11 (42)	43 ± 13 (42)
LVEDV (ml)	152 ± 37 (146)	154 ± 39 (141)	152 ± 35 (150)
LVESV (ml)	54 ± 22 (50)	54 ± 21 (51)	53 ± 23 (50)
SV (ml)	98 ± 22 (96)	98 ± 24 (96)	99 ± 20 (97)
LVEF (%)	65 ± 8 (65)	65 ± 8 (65)	66 ± 8 (65)
CO (l/min)	6 ± 2 (6)	6 ± 2 (6)	6 ± 2 (5)
LVM (g)	161 ± 56 (156)	177 ± 61 (172)	141 ± 42 n
LGE presence	57 (57%)	57 (100%)	0

Data is represented as n (%) or mean ± SD (median). *BMI* body mass index, *CO* cardiac output, *LV* left ventricle, *LVEF* ejection fraction, *LVEDV* end-diastolic volume, *LVESV* end-systolic volume, *LVM*  LV mass, *LGE*  late gadolinium enhancement, *SV*  stroke volume

**Fig. 3:**
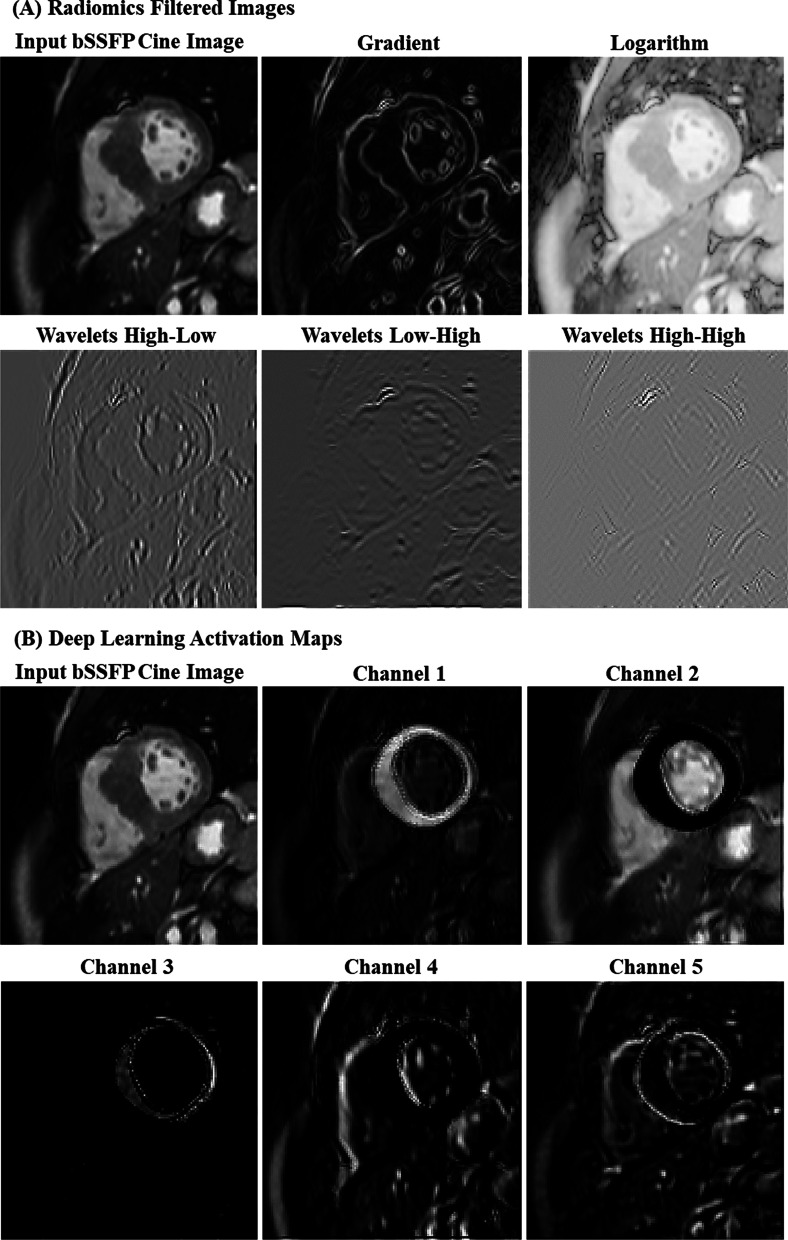
Initial processing of a non-gadolinium balanced steady state free precession (bSSFP) cine image for predicting myocardial scars. **A** Five preprocessed images (using 5 different image filters) used for radiomics feature extraction; **B** Five activation maps (from 5 different channels) generated by the first convolutional neural network (CNN) layer in the deep learning (DL) model

Visual inspection of the images pre-processed for radiomics analysis and those processed in the initial layer of the CNN network revealed significant differences (Fig. 3). DL showed ability to process regions within or outside the myocardium differently. For example, in channel 2 (Fig. 3), the myocardium was blurred while the edges of the extra-myocardial structures were enhanced.

**Fig. 4:**
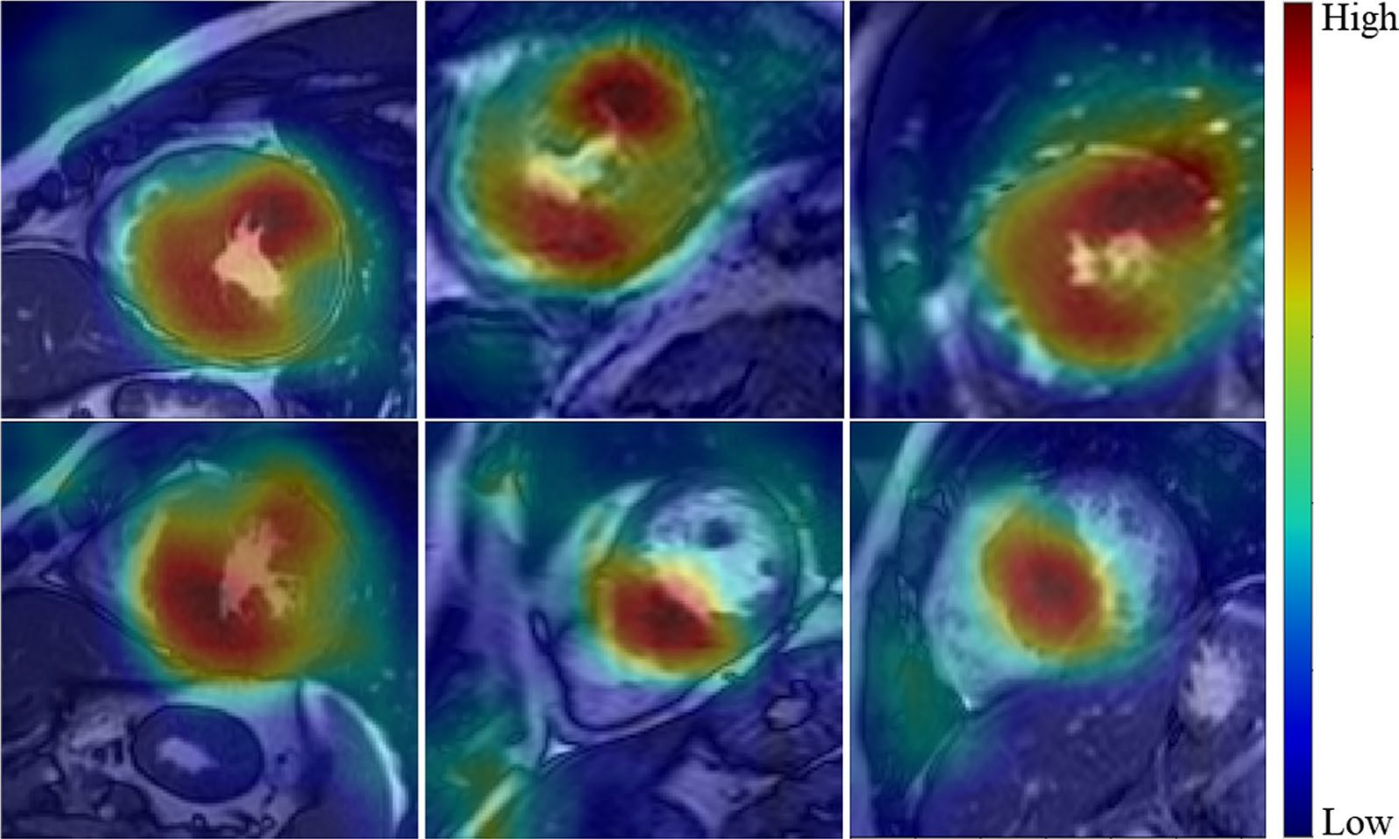
Heatmaps for six non-gadolinium bSSFP cine images (from 6 different patients with myocardial scar) displaying the importance of different image regions to the network decision of identifying myocardial scars

In the internal testing dataset, the Radiomics, DL, and DL-Radiomics models (trained using five different cross-validations) accurately identified patients without scar with AUC = 0.75 ± 0.03, 0.76 ± 0.01, and 0.81 ± 0.02, respectively (Table 3). The DL-Radiomics models showed consistently higher AUC and specificity than those of DL and Radiomics models alone in the majority of cross-validations (i.e., data-splits). At sensitivity ≥ 0.90, the DL-Radiomics model showed a higher average specificity (0.42 ± 0.06) compared to DL (0.40 ± 0.06) and Radiomics (0.36 ± 0.05) models. Of a note, a specificity of 0.42 means that only 42% of patients without scar were correctly identified and thus the necessity of LGE scans for this subset of patients may be reconsidered. To select a set of final models (i.e., DL, Radiomics, and DL-Radiomics models) for predicting scar in the external testing dataset, we selected the model with maximum AUC over all cross-validations. Among the selected models, DL-Radiomics model showed the highest AUC (0.83) compared to Radiomics (0.77, p = 0.006) and DL (0.78, p = 0.05) models. In the external testing dataset, all models showed lower performance compared to the internal dataset (Table 4). The DL-Radiomics model showed higher AUC (0.74) compared to both Radiomics (0.64, p = 0.006) and DL (0.71, p = 0.27) models (Table 4). Twenty-nine cases (28 false positive and 1 false negative) were misclassified by all three models, where imaging artifacts was observed in 16 cases (Additional file 1: Fig. S1). At sensitivity ≥ 0.90, DL-Radiomics model correctly identified 28% of patients without scar, which was higher compared to DL model (23%) and Radiomics model (16%) (Table 4). Visualization of the contribution of different myocardial regions to the DL decision of identifying a scar revealed that the septum is the most influential region common to almost all cases with scar (Fig. 4). Also, lateral myocardial segments showed important contributions to DL classification in addition to septal regions but not alone.

**Table 3:** Performance of the scar prediction models trained using 5 different splits of the development dataset

Cross-validation		1	2	3	4	5	Mean ± SD
Radiomics	Sensitivity	0.92	0.91	0.92	0.91	0.91	0.91 ± 0.01
	Specificity	0.42	0.36	0.37	0.37	0.29	0.36 ± 0.05
	Recall	0.85	0.81	0.84	0.82	0.77	0.82 ± 0.03
	Precision	0.58	0.56	0.57	0.56	0.53	0.56 ± 0.02
	Accuracy	0.65	0.62	0.63	0.62	0.58	0.62 ± 0.03
	AUC	0.77	0.76	0.77	0.72	0.71	0.75 ± 0.03
Deep learning	Sensitivity	0.92	0.92	0.91	0.92	0.91	0.92 ± 0.01
	Specificity	0.45	0.42	0.42	0.42	0.30	0.40 ± 0.06
	Recall	0.86	0.85	0.83	0.85	0.78	0.83 ± 0.03
	Precision	0.60	0.58	0.58	0.58	0.54	0.58 ± 0.02
	Accuracy	0.67	0.65	0.65	0.65	0.58	0.64 ± 0.03
	AUC	0.76	0.76	0.75	0.78	0.77	0.76 ± 0.01
Deep learning–radiomics	Sensitivity	0.91	0.91	0.91	0.91	0.91	0.91 ± 0.00
	Specificity	0.43	0.45	0.43	0.49	0.32	0.42 ± 0.06
	Recall	0.84	0.84	0.84	0.85	0.79	0.83 ± 0.02
	Precision	0.59	0.60	0.59	0.61	0.54	0.59 ± 0.03
	Accuracy	0.65	0.67	0.65	0.69	0.60	0.65 ± 0.03
	AUC	0.79	0.82^*†^	0.83^*†^	0.82^*^	0.79^*^	0.81 ± 0.02

Performance is evaluated using the internal testing dataset

^*^Statistical significance vs Radiomics model.

^†^Statistical significance vs DL model. All metrics were computed at an operating point corresponding to a sensitivity of at least 90%

**Table 4:** Performance of the final models using radiomics, deep learning (DL), and combined DL-Radiomics features for detecting scar in non-gadolinium cine images in the external cohort (n = 100 patients)

	AUC	Accuracy	Sensitivity	Specificity	Recall	Precision
Radiomics	0.64	0.59	0.91	0.16	0.58	0.59
Deep learning	0.71	0.62	0.91	0.23	0.67	0.61
DL-radiomics	0.74^*^	0.64	0.91	0.28	0.71	0.63

*AUC*  area under receiver operating curve

^*^Statistical significance vs radiomics model

## Discussion

We demonstrated that post-processing of bSSFP cine images has the potential to identify HCM patients without myocardial scar, in whom administration of GBCA can be avoided. The bSSFP signal intensity encodes myocardium tissue properties and is weighted by T_2_/T_1_ and magnetization-transfer (MT) [32], which are perturbed in presence of scar. However, visual assessment of bSSFP images cannot be reliably used to identify subtle intensity variations of scarred regions. In this study, we hypothesized that radiomics and DL features discriminate between healthy and scarred myocardium. To test this hypothesis, we developed and compared three AI-based scar prediction models based on three types of bSSFP cine image features: Radiomics, DL, and combined DL-Radiomics. In the internal testing dataset, all models showed potential in discriminating patients with and without scar (AUC > 0.75). DL-Radiomics models consistently showed higher performance compared to DL or Radiomics models in both internal and external datasets. Although the DL models were optimized to extract cine image features most important for scar detection, adding radiomic features improved the performance. However, there can be other DL architectures that allow learning a higher level of image representation that can replace all radiomic features.

In contrast to diagnostic applications, where disease identification models need to operate at high accuracy, we set our AI-models to operate at a high sensitivity (≥ 0.90) to minimize misclassifying patients with scar. Operating the models at high sensitivity thresholds substantially reduced their specificity and accuracy. However, specificity represents the gained ratio of patients without scar who are correctly identified by the model and for whom LGE scans may be avoided. In the internal dataset, all models showed approximately the same specificity (~ 42%). However, in the external dataset, the specificity of the DL-Radiomics model dropped from 0.42 to 0.28 but was still higher than DL (0.23) or Radiomics (0.16) models. There was a drop in the performance metrics of all models in the external dataset, which can currently limit their clinical use and suggests the need for further improvements in the generalizability of the models. Imaging artifacts in the external dataset (Additional file 1: Fig. S1) can be a reason for low model performance, however not all failure cases had image artifacts. We also note that limited generalizability is common to all data-driven models. Improving the external utility of artificial intelligence (AI) models may be achieved through a number of techniques [33–35] and should be further investigated.

The developed image analysis pipeline is comprised of two feature extraction modules: DL and radiomics. Although computing the DL features was much faster compared to radiomics (7 ms vs 1 s per image; on DGX-1 AI workstation (Nvidia, Santa Clara, California, USA)), the difference in speed is not a bottleneck of adopting either of these approaches. The post-processing of bSSFP cine can be performed during the time interval between short axis bSSFP cine and contrast administrations, which typically takes several minutes. Both approaches rely on segmentation of myocardial tissue, which can be automatically performed inline. Further studies are warranted to explore the feasibility of integrating such approach inline.

Clinical interpretation of features and AI model is an important and challenging aspect of AI research. Several of radiomic features have clinical interpretations. For example, LV geometric descriptors such as maximum diameter and elongation are directly related to LV dilation and sphericity, respectively and the image intensity descriptors (e.g., minimum value and summation) are related to the T_1_/T_2_ properties of the myocardium. Nevertheless, it is challenging to interpret all radiomic or DL features due to the highly nonlinear relationship between these features and the myocardium image intensities. Radiomics models can be more interpretable than DL models because radiomic features represent well-defined mathematical quantities. However, it is challenging to identify how radiomic feature values change with tissue properties. Furthermore, DL models involve highly nonlinear processing of the input images, which does not allow explanation of the model outputs. We used grad-cam to visualize the cine image regions contributed most to the DL model predictions and observed that the septal and lateral myocardial segments contributed to the model predictions. However, it is premature to use these observations to draw further conclusions regarding the image features driving the model predictions. In addition, there remains controversies on the trustworthiness of model interpretation techniques [36]. Further analysis of the model interpretation may be needed to identify the reasoning for model behavior.

We observed that only a few radiomic features were common among different cross-validations in the Radiomics model (Additional file 2: Table S2) or the DL-Radiomics model (Additional file 2: Table S3). Despite differences in important radiomics features between different cross-validation splits, the performance was consistent across the different cross-validation splits, which is expected given the high correlation among the radiomic features [37, 38]. We also observed similar differences between the selected important radiomic features between the DL-Radiomics and Radiomics models. The improved performance of the DL-Radiomics model potentially suggests that DL and radiomic features carry different myocardial texture properties. Further investigations are needed to determine the relationship between the DL and radiomic features.

Although current model performance is not yet adequate for clinical deployment, it shows the potential of AI in unmasking hidden information in native bSSFP cine images. Further improvements of AI-based screening models for myocardial scarring can be achieved by exploiting additional information from other sequences such as T_1_ or T_2_ mapping, and myocardial strain. Incorporating clinical variables into the model may also provide additional improvement in the model performance. However, further technical developments to overcome current limitations such as image registration are needed to fully take advantages of additional imaging and clinical markers. Due to retrospective nature of our study, our dataset did not contain T_1_ or T_2_ mapping images, therefore, we were not able to explore their potential in improving model performance.

### Limitations

Our study has several limitations. First, we did not test the reproducibility of the radiomics features with different manual segmentations. There are differences in repeatability and reproducibility of among different radiomics features, which can be sensitive to image acquisition parameters and likely impacted by vendors and field strength [37–40]. We only included patients with known HCM patients and did not study the performance of the model in other non-ischemic cardiomyopathy patients.

## Conclusions

A combined DL-Radiomics AI model using bSSFP cine images outperforms the DL or Radiomics models alone in identifying HCM patients without scar as a screening tool for gadolinium administration. The developed AI models have potential to identify HCM patients without scar who may be triaged from unnecessary gadolinium administration. Despite potential of such screening model, clinical utility remains limited and further investigation is needed to improve the accuracy and generalization.

## Supplementary Information

**Additional file 1: Figure S1**. Example cine slices from cases with failed scar predictions by all three models.

**Additional file 2: Table S1.** Performance of the different machine learning classifiers used for predicting scar from 20 deep learning and radiomic features. Performance was measured by the area under receiver operator curve (AUC) averaged over 5-fold cross-validations of the development dataset. **Table S2.** List of the most important radiomics features selected for scar prediction using Radiomics-only models. **Table S3.** List of the most important radiomics features selected for scar prediction using combined Deep Learning-Radiomics model.

## Data Availability

The datasets analyzed during the current study are not publicly available due to patient privacy constrains.

## Abbreviations

AI: Artificial intelligence
bSSFP: Balanced steady state free precession
CMR: Cardiovascular magnetic resonance
CNN: Convolutional neural network
DL: Deep learning
GBCA: Gadolinium based contrast agent
HCM: Hypertrophic cardiomyopathy
LGE: Late gadolinium enhancement
LV: Left ventricle/left ventricular
LVEDV: Left ventricular end-diastolic volume
LVEF: Left ventricular ejection fraction
LVESV: Left ventricular end-systolic volume
LVM: Left ventricular mass
