# Supplementary figures and images for: Radiomics and deep learning for myocardial scar screening in hypertrophic cardiomyopathy
Source: J Cardiovasc Magn Reson. 2022 Jun 27;24:40. doi: 10.1186/s12968-022-00869-x (PMC9235098; doi:10.1186/s12968-022-00869-x)

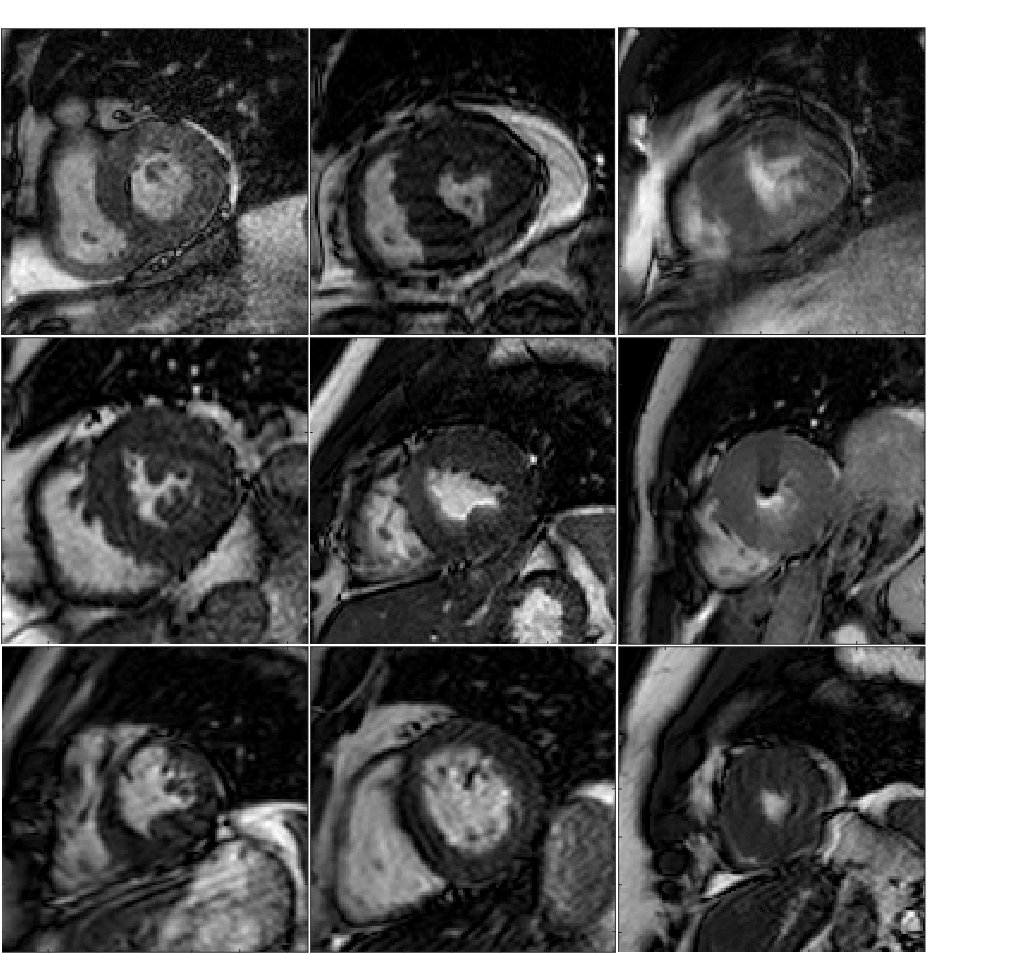


**Figure S1**. Example cine slices from cases with failed scar predictions by all three models.”

Supplement: Supplementary file 1 — Additional file 1: Figure S1. Example cine slices from cases with failed scar predictions by all three models. [file 12968_2022_869_MOESM1_ESM.docx]
