# Supplementary material for: Radiomics and deep learning for myocardial scar screening in hypertrophic cardiomyopathy
Source: J Cardiovasc Magn Reson. 2022 Jun 27;24:40. doi: 10.1186/s12968-022-00869-x (PMC9235098; doi:10.1186/s12968-022-00869-x)
Supplement: Supplementary file 2 — Additional file 2: Table S1. Performance of the different machine learning classifiers used for predicting scar from 20 deep learning and radiomic features. Performance was measured by the area under receiver operator curve (AUC) averaged over 5-fold cross-validations of the development dataset. Table S2. List of the most important radiomics features selected for scar prediction using Radiomics-only models. Table S3. List of the most important radiomics features selected for scar prediction using combined Deep Learning-Radiomics model. [file 12968_2022_869_MOESM2_ESM.docx]

**Table S.1** Performance of the different machine learning classifiers used for predicting scar from 20 deep learning and radiomic features. Performance was measured by the area under receiver operator curve (AUC) averaged over 5-fold cross-validations of the development dataset.

| **AUC** | **Random Forests** | **Gradient Boosted Trees** | **Support Vector Machines** | **4-layer Neural Networks**  **(#nodes: 32,16,4,1)*** | **Logistic Regression** |
| --- | --- | --- | --- | --- | --- |
| **Per-Patient** | 0.83±0.05 | 0.80±0.06 | 0.81±0.06 | 0.83±0.05 | 0.83±0.04 |
| **Per-Slice** | 0.81±0.06 | 0.78±0.06 | 0.80±0.06 | 0.81±0.05 | 0.81±0.05 |

* The neural network model with the best performance among different models with different architectures (number of layers from 1 to 5, number of nodes from to 20 to 100).

**Table S.2** List of the most important radiomics features selected for scar prediction using Radiomics-only models.

| **Rank** | **Cross-**  **Validation 1** | **Cross-**  **Validation 2** | **Cross-**  **Validation 3** | **Cross-**  **Validation 4** | **Cross-**  **Validation 5** |
| --- | --- | --- | --- | --- | --- |
| **Shape Descriptors** | - LV Max Diameter | - LV Max Diameter | - LV Max Diameter | - LV Max Diameter | - LV Max Diameter |
| **Intensity values** | - EX-Min Intensity | - EX-Min Intensity - SR-Energy - WV/LL-10 Percentile | - EX-Min Intensity - SR-Energy | - EX-Min Intensity - SR-10 Percentile - SR-Energy | - EX-Min Intensity - SR-10 Percentile - SR-Energy |
| **Wavelets Texture Descriptors** | - LH-GLCM- Correlation - LH-GLCM-IMC2 - HL-GLRLM-GLNU - HL- GLRLM-RunEntropy | - LH-GLCM- Correlation - LH-GLCM-IMC2 - HL-GLRLM-GLNU - HL-GLRLM-RunEntropy | - LH-GLCM- Correlation - LH-GLCM-IMC2 - HL-GLRLM-GLNU - HL- GLRLM-RunEntropy | - LH-GLCM-IMC2 - HL- GLRLM-RunEntropy - LL-GLCM-Joint Average | - LH-GLCM-IMC2 - HL- GLRLM-RunEntropy |
| **Other Texture Descriptors** | - EX-GLCM- Correlation, - EX-GLCM-IDN - GR-GLRLM-GLNU - SQ-GLCM-IMC1 | - EX- GLCM- Correlation - GR-GLRLM-GLNU | - EX-GLCM- Correlation - GR-GLRLM-GLNU - SQ-GLCM-IMC1 | - OG-GLCM-IMC1 - OG-NGTDM- Busyness - EX-GLDM-GLNU | - OG-NGTDM- Busyness - OG-GLCM- Joint Average - EX-GLDM-GLNU - LG-GLCM-IDMN |

EX= exponential transformed image

GLCM = gray-level co-occurrence matrix

GLDM = gray level dependence matrix

GLNU = gray-level non-uniformity

GLRLM = gray-level run-length matrix

HL = high-low pass filtered image (wavelet transform)

IDMN = inverse difference moment normalized

IDN = inverse difference normalized

IMC = informational measure of correlation (type 1 or 2)

LG = logarithmic transformed image

LH = low-high pass filtered image (wavelet transform)

LL = low-low pass filtered image (wavelet transform)

LV = Left Ventricle

NGTDM = neighboring gray tone difference matrix

OG = Original input image (i.e. without transformation)

RunEntropy = run-length entropy

SQ = squared image

SR = square-root transformed image

WV = wavelet transformed image

**Mathematical formulae of the radiomics features listed above can be found in**

<https://pyradiomics.readthedocs.io/en/latest/features.html#radiomic-features>

**Table S.3** List of the most important radiomics features selected for scar prediction using combined Deep Learning-Radiomics model.

| **Rank** | **Cross-**  **Validation 1** | **Cross-**  **Validation 2** | **Cross-**  **Validation 3** | **Cross-**  **Validation 4** | **Cross-**  **Validation 5** |
| --- | --- | --- | --- | --- | --- |
| **Shape Descriptors** | - LV Max Diameter | - LV Max Diameter - LV Elongation | - LV Max Diameter | - None | - LV Max Diameter - LV Elongation |
| **Intensity Statistics** | - SR-Energy - LL-10 Percentile | - OG-Energy - LL-Energy | - SR-Energy - EX-Min Intensity | - SR-Energy | - EX-Min Intensity - EX-Skewness - LL-Energy |
| **Wavelets Texture Descriptors** | - LH-GLCM-IMC2 - HL-GLRLM-RunEntropy - LL-GLCM-SumEntropy | - LH-GLCM-Contrast - LH-GLCM-IMC2 - HL-GLDM-normalized DNU - HL-GLRLM-RunEntropy | - LH-GLCM-Correlation - LH-GLCM-IMC2 - HL-GLDM- normalized DNU - HL-GLRLM-GLNU - HL-GLRLM-RunEntropy | - LH-GLCM-Contrast - LH-GLCM-IMC2 - HL-GLRLM-GLNU - HL-GLRLM-RunEntropy | - LH-GLCM-IMC2 - HL-GLRLM-GLNU - HL-GLRLM-RunEntropy - LL-GLCM-Correlation |
| **Other Texture Descriptors** | - SQ-GLDM-DNU - SR-GLDM- LgDepHiGL | - OG-GLCM-Contrast - OG-GLCM-MCC - OG-NGTDM-Busyness - GR-GLRLM-GLNU - SQ-GLDM-DNU | - EX-GLCM-Idn - SQ-GLCM-Difference Average - SQ-GLCM-IMC1 - SQ-GLDM-DNU | - OG-GLCM-MaxProb - EX-GLCM-CT - SQ-GLDM-DNU - SR-GLCM-MCC | - OG-GLCM- Correlation - OG-GLCM-MCC - OG-NGTDM-Busyness - EX-GLCM-Idn - SQ-GLDM-DNU - SQ-GLDM-GLNU |
| **Deep Learning Features** | - 12 features | - 7 features | - 8 features | - 11 features | - 5 features |

CT = ClusterTendency

DNU = Dependence NonUniformity

EX= exponential transformed image

GLCM = gray-level co-occurrence matrix

GLDM = gray level dependence matrix

GLNU = gray-level non-uniformity

GLRLM = gray-level run-length matrix

HL = high-low pass filtered image (wavelet transform)

IDMN = inverse difference moment normalized

IDN = inverse difference normalized

IMC = informational measure of correlation (type 1 or 2)

LG = logarithmic transformed image

LgDepHiGL = Large Dependence High GrayLevel Emphasis

LH = low-high pass filtered image (wavelet transform)

LL = low-low pass filtered image (wavelet transform)

LV = Left Ventricle

NGTDM = neighboring gray tone difference matrix

OG = Original input image (i.e. without transformation)

RunEntropy = run-length entropy

SQ = squared image

SR = square-root transformed image

WV = wavelet transformed image
